# Root Branching Is a Leading Root Trait of the Plant Economics Spectrum in Temperate Trees

**DOI:** 10.3389/fpls.2017.00315

**Published:** 2017-03-08

**Authors:** Rebecca Liese, Katrin Alings, Ina C. Meier

**Affiliations:** Plant Ecology, Albrecht-von-Haller Institute for Plant Sciences, University of GöttingenGöttingen, Germany

**Keywords:** angiosperm trees, arbuscular mycorrhiza, ectomycorrhiza, fine root traits, gymnosperm trees, precision foraging, root economics spectrum, root order

## Abstract

Global vegetation models use conceived relationships between functional traits to simulate ecosystem responses to environmental change. In this context, the concept of the leaf economics spectrum (LES) suggests coordinated leaf trait variation, and separates species which invest resources into short-lived leaves with a high expected energy return rate from species with longer-lived leaves and slower energy return. While it has been assumed that being fast (acquisitive) or slow (conservative) is a general feature for all organ systems, the translation of the LES into a root economics spectrum (RES) for tree species has been hitherto inconclusive. This may be partly due to the assumption that the bulk of tree fine roots have similar uptake functions as leaves, despite the heterogeneity of their environments and resources. In this study we investigated well-established functional leaf and stature traits as well as a high number of fine root traits (14 traits split by different root orders) of 13 dominant or subdominant temperate tree species of Central Europe, representing two phylogenetic groups (gymnosperms and angiosperms) and two mycorrhizal associations (arbuscular and ectomycorrhizal). We found reflected variation in leaf and lower-order root traits in some (surface areas and C:N) but not all (N content and longevity) traits central to the LES. Accordingly, the LES was not mirrored belowground. We identified significant phylogenetic signal in morphological lower-order root traits, i.e., in root tissue density, root diameter, and specific root length. By contrast, root architecture (root branching) was influenced by the mycorrhizal association type which developed independent from phylogeny of the host tree. In structural equation models we show that root branching significantly influences both belowground (direct influence on root C:N) and aboveground (indirect influences on specific leaf area and leaf longevity) traits which relate to resource investment and lifespan. We conclude that branching of lower order roots can be considered a leading root trait of the plant economics spectrum of temperate trees, since it relates to the mycorrhizal association type and belowground resource exploitation; while the dominance of the phylogenetic signal over environmental filtering makes morphological root traits less central for tree economics spectra across different environments.

## Introduction

Plant functional trait spectra are valuable tools in simplifying floristic complexity to a level that can be handled in models which scale ecosystem processes to landscape and global scales. Theory on plant growth strategies suggests that plants characteristic of low- and high-resource environments, respectively, evolved a common set of traits linking exploitation (root:shoot, tissue turnover, and concentration of plant defences) with growth (resource uptake and growth rates; Grime, 1977; Chapin et al., 1993; Bardgett et al., 2014). In continuation of this theory, the leaf economics spectrum (LES) describes a universal spectrum on the return of nutrient and dry mass investments in leaves (Wright et al., 2004): fast, acquisitive species with high expected rate of energetic return on investment possess relatively large, fast growing leaves with short lifespan, high N content per unit mass, high specific leaf area (SLA), and high instantaneous rates of respiration and photosynthesis in comparison to slow species. This suggests convergence of leaf traits of coexisting species under similar environmental conditions, despite the great genotypic diversity among these species (Reich et al., 2003). The LES seems to operate largely independent of growth form, plant functional type, or biome (Wright et al., 2004), and has been successfully linked to plant performance (Reich et al., 1998; Poorter and Bongers, 2006), species distribution and interactions (Sterck et al., 2006), and ecosystem processes and services (Reich et al., 1997; Díaz et al., 2004; Grigulis et al., 2013; Weemstra et al., 2016).

Despite the successful application of the LES and the translation into a correspondent wood economics spectrum (WES; Chave et al., 2009), its translation into a root economics spectrum (RES) for trees has been inconclusive so far and is still a matter of debate. By theory, being fast or slow should be a general feature of species (Reich, 2014). Consequential, acquisitive species with respect to their leaf traits should possess relatively small-diameter, fast-growing fine roots with short lifespan, high N content, high specific root length (SRL), and high rates of respiration and nutrient acquisition in comparison to slow, conservative species with long-term resource retention. This theoretic RES has been partly confirmed for trees in some studies (Chen et al., 2010; McCormack et al., 2012; Reich, 2014), but scrutinized by others (Comas and Eissenstat, 2004; Withington et al., 2006; Chen et al., 2013; Valverde-Barrantes et al., 2015; Weemstra et al., 2016). Often, not the whole set of traits for a RES for mature trees is covered by single studies using standardized methods, which makes overall conclusions difficult.

The complex architecture of root systems has traditionally been categorized according to root diameter in fine and coarse roots, which may not reflect their functionality, especially among tree species with systematic differences in mean root diameter. More recent work, which focused on the classification of fine roots according to a stream-based ordering system (Pregitzer et al., 2002), has proved that only the most distal fine root orders serve (primarily) water and nutrient acquisition (Guo et al., 2008; Rewald et al., 2011; McCormack et al., 2015). These distal fine root orders should have similar functionality across species and be a reflection of the resource acquisition function of leaves, which makes their traits more suitable for an inspection of the RES. However, resource uptake belowground differs vastly from aboveground resource capture: light and CO_2_ are predictably available throughout the canopy while nutrients and water are often highly heterogeneously distributed in the soil, which increases the importance of traits related to precision foraging (prolific root branching and mycorrhizal symbioses) over traits which maximize the surface area *per se*. The branching architecture of roots is an expression of the plastic responses to their environment since it seems to be independent from phylogeny, at least in subtropical trees (other than diameter-related root traits; Kong et al., 2014). It has been demonstrated that species with high branching intensity are capable of rapid and extensive proliferation into resource-rich patches (morphological plasticity; reviewed by Hodge, 2004). Traits related to precision foraging of roots are missing in the current version of the RES, though (Weemstra et al., 2016). In particular the association with mycorrhizal fungi may complement the foraging strategy of roots for limiting nutrients. Trees associated with different mycorrhizal colonization types differ profoundly in root traits related to precision foraging: ECM trees, which mainly occur in ecosystems dominated by organic nutrients, have thinner roots and higher branching intensity than AM trees (Brundrett, 2002; Smith and Read, 2008; Comas and Eissenstat, 2009; Comas et al., 2014; Eissenstat et al., 2015). Yet it is unknown if ECM trees belong systematically to the more acquisitive root spectrum in comparison to AM trees.

In the work presented here, we analyzed sun leaf, stature, and fine root traits of the first to fifth root order of thirteen important temperate tree species of the Central European tree flora, which represented two phylogenetic groups (gymnosperms and angiosperms) and two mycorrhizal association types (AM and ECM). Sun leaf and fine root samples were collected from three mixed forest stands in the center of Germany. For the comparison of fine roots which serve similar functions among tree species, we separated fine root strands into two root order fractions (first to second and first to fifth root orders). We analyzed fine root fractions for thirteen traits, including specific root area (SRA), SRL, tissue density, branching ratio, branching intensity, root diameter, root N_mass_, and root C:N, and obtained information on fine root longevity from an accompanying comprehensive literature survey. We hypothesized that (i) fine root morphology is phylogenetically structured, (ii) the RES is not a mirrored analogy of the LES, but centers around traits related to precision foraging, i.e., around root branching, in which trees with intense root branching belong to the fast, acquisitive spectrum and trees with reduced root branching belong to the slow, conservative spectrum, and (iii) ECM trees have higher branching intensity and more acquisitive root traits in comparison to AM trees.

## Materials and Methods

### Study Sites and Tree Species

Sampling from 13 major Central European tree species was conducted in three mixed forest stands in Central Germany, which represented characteristic, mesic mesotroph site conditions for the investigated tree species: two study sites incorporated replicate sites for angiosperm tree species (‘Hainich National Park’ at 340 m a.s.l., 51°08′N, 10°51′E and ‘Experimental Botanical Garden Göttingen’ at 200 m a.s.l., 51°55′N, 9°96′E) and one study site covered the gymnosperm tree species (‘Moringen City Forest’ at 310 m a.s.l., 51°73′N, 9°86′E). Stands were mature and even-aged, and predominately hardwoods (or hardwoods interspersed with evergreens in the case of the Moringen City Forest). All sites had a mean annual temperature between 7.5 and 9.0°C and mean annual precipitation between 630 and 670 mm. Last forest management activities occurred at least a couple of decades ago and soil manipulation activities such as liming were absent.

The selected major tree species of the Central European forest flora are either dominant species of the natural forest vegetation or are frequently present in forest communities as subdominant or admixed species. The 13 species represent a broad range of taxa, covering eleven genera, eight families, and six orders (Supplementary Table 1). Among the 13 species are four conifers (family *Pinaceae*) and nine deciduous broad-leaved species from the families *Fagaceae, Sapindaceae, Malvaceae, Betulaceae, Oleaceae*, and *Rosaceae*. The species were selected to represent two phylogenetic groups (gymnosperms and angiosperms) and two mycorrhizal association types (AM and ECM; Supplementary Table 1). The association to a mycorrhizal association type was assigned to according to literature (Wang and Qiu, 2006), and was confirmed by measurements of the arbuscular and ectomycorrhizal colonization rates in an accompanying study (Liese, pers. communication).

### Leaf and Fine Root Sampling and Analyses

Leaf samples of angiosperm tree species were collected from the upper sun canopy with the help of canopy walkways in mid-summer 2014 (*n* = five leaf samples each of five individuals per tree species and study site). Leaf samples were stored at 6°C for no more than a week until processing. All leaves were analyzed for leaf area using a flat-bed scanner and the computer program WinFOLIA (2005b; Régent Instruments Inc., Canada). Subsequently, the total leaf mass was dried (70°C, 48 h) and weighed and the SLA (cm^2^ g^-1^) calculated. Dried leaf samples were ground and total carbon and nitrogen content analyzed using a C/N elemental analyzer (vario EL III, elementar, Hanau, Germany). Sun leaf samples of gymnosperm trees were not easily accessible and trait information was derived from a comprehensive literature survey instead (see below; Supplementary Table 2).

Fine root samples of all tree species were carefully excavated from the uppermost 20 cm of the soil profile in close surroundings (<50 cm) of mature canopy trees of the respective species, which were growing in single-species tree clusters, and were traced toward their mother tree (*n* = 10 root samples each of at least five different individuals per tree species and study site). Root samples were immediately transported to the laboratory and stored moist at 6°C for no longer than 3 weeks until processing. Root strands were cleared from soil particles with tap water and the tree species identity was confirmed a second time under a stereomicroscope (magnification × 40) with a site-specific morphological key based on periderm structure and color, root ramification, and root tip morphology (*cf.* Meinen et al., 2009; Kubisch et al., 2015). All vital, intact root strands were cut at the end of the fifth root order (stream-based ordering system according to Pregitzer et al., 2002, with the most distal root segments being classified as first root order) for comparability between tree species. We selected to cut root systems at the end of the fifth root order, since the sixth and higher order roots occasionally comprised roots with a diameter >2 mm, i.e., could not be classified as fine roots. The first to fifth root orders were constituted of only fine roots (diameter <2 mm) in all investigated tree species. We counted root tips of these intact root systems under a stereomicroscope.

Half of the intact root samples were analyzed for their morphology of the first to fifth root order using a flat-bed scanner and the computer program WinRHIZO (2005c; Régent Instruments Inc., Canada; resolution: 200 dpi; *n* = five root samples each of at least five different individuals per tree species and study site) in order to determine root length, surface area, diameter, and volume. Root systems comprising the first to fifth root order were analyzed intact for comparability with other studies that are not separating between different root orders. Subsequently, root strands were dissected with scalpels under a stereomicroscope to separate the absorbing root orders, i.e., the first and second order (Guo et al., 2008; Valenzuela-Estrada et al., 2008) from the transport root orders, i.e., third to fifth order. Dissected first and second root orders were scanned again and analyzed for their morphology. The two root order fractions (first and second order and third to fifth order) were dried (70°C, 48 h) and weighed. SRA (cm^2^ g^-1^), SRL (cm g^-1^), tissue density (g cm^-3^), and mean root diameter were calculated independently for (i) the first and second root order and (ii) the first to fifth root order. The branching ratio was determined from the number of first order roots growing out of second order roots (n n^-1^). Branching intensity was calculated from the number of root tips per root length of first and second order roots (tips cm^-1^). The absorptive to transport root ratio was calculated by dividing the mass of the first and second root orders by the mass of the third to fifth root orders (g g^-1^).

The second half of the intact root samples was dried (70°C, 48 h), ground, and total carbon and nitrogen content analyzed using a C/N elemental analyzer (vario EL III, elementar, Hanau, Germany; *n* = five root samples each of at least five different individuals per tree species and study site). The analyzed C:N_1-5_ describes the C/N ratios of a representative fine root population for all tree species, comprising the first to fifth root order.

### Additional Traits

Based on a comprehensive literature survey and additional data (SLA, leaf N, and maximum tree height) from the TRY Plant Trait Database (Kattge et al., 2011), we assembled a database of about 40 published and unpublished studies that contained information related to SLA and leaf N (for the four gymnosperms of interest to this study), as well as information on leaf longevity, maximum tree height, wood density, maximum tree age, and fine root longevity (for all 13 tree species of interest to this study). Selection criteria for data were (a) study plot located in the cool-temperate zone of Central Europe, (b) measurements taken in mature trees (>40 years old) in monospecific stands with closed canopy, (c) last forest management activities occurred at least a decade ago, and (d) absence of soil manipulation such as liming. All data on SLA referred to sun leaves in the upper sun canopy and mostly were taken using towers or cranes.

### Phylogenetic Signal

The phylogenetic signal was estimated by the correlation between the phylogenetic distance and trait distance matrices among the investigated tree species. We attached our list of taxa to the master phylogeny presented by Zanne et al. (2014) with the help of the software PHYLOMATIC v3 (a tool associated to PHYLOCOM 4.2; Webb et al., 2008), to generate the initial phylogenetic tree in the Newick format. The simple pairwise matrix of phylogenetic distances was calculated from the Newick code with the ‘phydist’ phylogeny tool in PHYLOCOM and visualized with the online tool iTOL – Interactive Tree Of Life v3.1 (Ciccarelli et al., 2006; Supplementary Figure 1).

We identified major trait complexes explaining more than 75% of the variance for leaf, stature, and root traits, respectively, by calculating three independent PCAs, using the package Canoco 5.03 (Biometris, Wageningen University and Research Centre, The Netherlands; Supplementary Table 3). Independent trait distance matrices based on the PCA axes for leaf, stature, and root traits, respectively, were calculated with the package SAS, version 9.3 (Statistical Analyses System, SAS Institute Inc., Cary, NC, USA). For the correlation between the phylogenetic and trait distance matrices, a Mantel permutation test (Mantel, 1967; Mantel and Valand, 1970) was computed with PAST 3.11 (Øyvind Hammer, Natural History Museum, University of Oslo, Norway), and the Pearson correlation coefficient *R* and the one-tailed *P*-value from the comparison of the original *R* to the *R* computed in 9999 random permutations were reported. Euclidean similarity indices were used for the Mantel permutation test.

As a second estimate of the phylogenetic signal, we conducted node-level analyses of traits and of trait conservation. We determined the average standard deviation of values at daughter nodes (‘divergence’) as a measure of trait radiation at this node (conservative: divergence <1, divergent: divergence >1) with the ‘aot’ phylogenetic trait analysis algorithm in PHYLOCOM (999 randomizations) and calculated the node age as branch length in percent of total phylogenetic distance.

### Statistical Analyses

All data were tested for probability of fit to normal distribution by a Shapiro–Wilk test (SAS 9.3; SAS Institute Inc., Cary, NC, USA). Leaf and root longevity were log-transformed to correct departures from normality. We tested for multicollinearity between traits by Pearson correlations and identified collinearity for the correlation between leaf C:N and leaf longevity, SRA_1+2_ and tissue density_1+2_, and SRL_1+2_ and tissue density_1+2_ (*R* > 0.90); all three were thus excluded from further analyses. Means of the tree groups (AM angiosperms, ECM angiosperms, and ECM gymnosperms) were compared by one-way analyses of variance (ANOVA) followed by a Scheffé test. Mixed variance-covariance models for fixed and random effects with the variables mycorrhizal association type (AM vs. ECM) and phylogenetic group (gymnosperm vs. angiosperm) were calculated to test for significant effects. Data likelihood was maximized to estimate the model parameters. A canonical correspondence analysis (CCA) was calculated for the stepwise forward selection of root traits that maximized the centroid distances between ECM gymnosperms, ECM angiosperms, and AM angiosperms, using the package Canoco 5.03 (Biometris, Wageningen University and Research Centre, The Netherlands). A total of 499 random permutations were used.

We used SPSS Amos 24.0.0 software (IBM, Somers, NY, USA) to calculate structural equation models (SEM). SEM was applied for identifying the direct and indirect effects of fine root branching intensity and branching ratio (as indication of the mycorrhizal association type) on leaf, stature, and fine root traits other than root branching intensity and branching ratio in the investigated tree species. We started with an initial model that contained all plausible interactions between root, stature, and leaf traits (Supplementary Figure 2). Path coefficients were determined as standardized regression weights using the maximum likelihood method. Modification indices were used to evaluate potential modifications of the model which were plausible and minimized the χ^2^. Two goodness-of-fit indices were accounted for [Tucker-Lewis Index TLI (Tucker and Lewis, 1973) and Root Mean Square Error of Approximation RMSEA (Browne and Cudeck, 1993)]. Insignificant paths were eliminated from the model. The square of the coefficient of multiple correlations *R*^2^ was calculated for all dependent variables.

## Results

### Above- and Belowground Trait Relations

**FIGURE 1:**
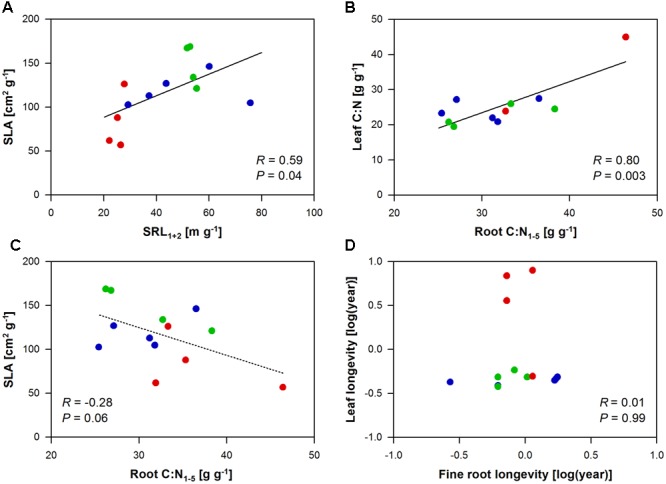
**Pearson’s correlation analyses between leaf and root traits of the ECM gymnosperm (red), ECM angiosperm (green), and AM angiosperm (blue) tree species analyzed in this study.** Given are the relationships between **(A)** SLA and SRL_1+2_, **(B)** leaf and root C:N_1-5_, **(C)** SLA and root C:N_1-5_, and **(D)** leaf and fine root longevity.

Specific leaf area, leaf C:N, and leaf longevity related to a number of root traits, while leaf N did not relate to any of the investigated root traits (Supplementary Table 4). SLA mainly correlated with the root morphology of the first and second root order (SRL_1+2_ and SRA_1+2_: positive correlation; diameter and tissue density: negative correlation), as well as with the branching intensity (i.e., the number of root tips per lower order root length; positive correlation) of the root system and its N content (positive correlation). In a direct comparison of the morphology of the absorbing tissues, SLA significantly increased by 12 cm^2^ g^-1^ with an increase in SRL_1+2_ by 10 m g^-1^ (**Figure 1A**). In an opposite trend, an increase in the C:N in the root tissue by 10 g g^-1^ correlated to a decrease in SLA by 32 cm^2^ g^-1^ (marginal significant; **Figure 1C**). Yet the strongest (positive) correlation with the root C:N had the leaf C:N ratio, which may hint to a whole plant trait coordination with respect to C:N variation (**Figure 1B**). Surprisingly, leaf longevity did not relate to root longevity neither in the whole tree species data set nor in the subset of angiosperm tree species (*P* = 0.99 and 0.38, respectively; **Figure 1D**). Leaf longevity was strongly positively correlated with root diameter and root tissue density_1+2_ (Supplementary Table 4).

### Phylogenetic Signal in Root and Leaf Traits

**FIGURE 2:**
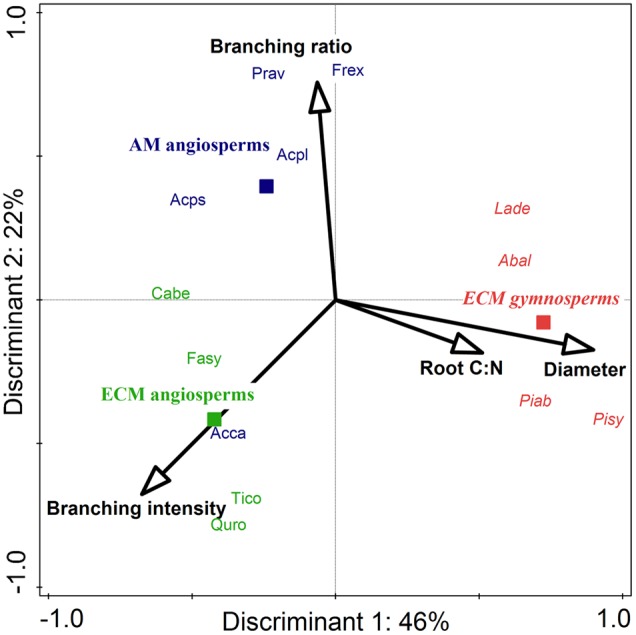
**Canonical correspondence analysis (CCA) for the stepwise selection of root traits for the discrimination between ECM gymnosperms (red italic), ECM angiosperms (green), and AM angiosperms (blue) among 13 Central European tree species.** Solid squares mark the centroid of each group. Out of a total of eight preselected root traits, four discriminants were needed to explain 65% of the variation, with the highest contribution by root diameter_1-5_ (*P* = 0.01) and branching intensity (*P* = 0.08). For abbreviations of tree species refer to Supplementary Table 1.

In a comparison of the two investigated phylogenetic groups (gymnosperms and angiosperms) it appears that there was a highly significant influence by phylogenetic group affiliation on the mean root diameters of all roots and on the root tissue densities of lower order roots: gymnosperms had a higher mean root diameter_1-5_ (0.53 vs. 0.39 mm) and a higher root tissue density_1+2_ (0.24 vs. 0.15–0.18 g cm^-3^) than angiosperm tree species (**Table 1**). Consequently, SRL_1+2_ of lower root orders (25 vs. 49–53 m g^-1^) and branching intensity (3.3 vs. 5.4–9.6 tips cm^-1^) of the gymnosperm root systems were reduced. Our discriminant analysis revealed that mean root diameter_1-5_ and root C:N_1-5_ were the most important root traits for the discrimination between gymnosperm and angiosperm tree species, and explained together 45% of the total variation (**Figure 2**).

**Table 1:** Trait values for AM angiosperm (*n* = 5), ECM angiosperm (*n* = 4), and ECM gymnosperm (*n* = 4) tree species (given are means and standard errors).

Traits	AM angiosperm	ECM angiosperm	ECM gymnosperm	CV [%]	Mycorrhizal association	Phylog. group
**Leaves**
SLA [cm^2^ g^-1^]	119 (8)ˆAB	148 (12)ˆA	83 (16)ˆB	30		^∗∗^
Leaf N_mass_ [mg g^-1^]	19 (1)	22 (1)	23 (4)	22		
Leaf C:N [g g^-1^]	24 (1)	22 (1)	36 (7)	27		^∗^
Leaf longevity^a^ [yr]	0.5 (0.02)ˆB	0.5 (0.04)ˆB	4.9 (1.7)ˆA	**149**		^∗∗^
**Stature**
Max. tree height^a^ [m]	34 (6)ˆB	48 (7)ˆAB	60 (6)ˆA	35		
Wood density^a^ [kg m^-3^]	598 (16)ˆAB	653 (62)ˆA	470 (32)ˆB	18		^∗∗^
Max. tree age^a^ [yr]	230 (44)ˆB	400 (54)ˆAB	413 (38)ˆA	37	^∗^	
**Roots**
SRL_1+2_ [m g^-1^]	49 (8)ˆA	53 (1)ˆA	25 (1)ˆB	38		^∗∗^
Tissue density_1+2_ [g cm^-3^]	0.18 (0.02)ˆB	0.15 (0.01)ˆB	0.24 (0.01)ˆA	23		^∗∗∗^
Branching ratio [n n^-1^]	2.8 (0.2)	2.3 (0.2)	2.5 (0.3)	19	(^∗^)	
Branching intensity [tips cm^-1^]	5.4 (1.2)ˆAB	9.6 (1.1)ˆA	3.3 (0.9)ˆB	**53**	^∗^	^∗∗^
Absorptive: transport roots [g g^-1^]	1.0 (0.3)	0.5 (0.1)	0.7 (0.2)	**55**	(^∗^)	
Root diameter_1+2_ [mm]	0.41 (0.02)	0.42 (0.01)	0.47 (0.01)	9		^∗^
Root diameter_1-5_ [mm]	0.39 (0.02)ˆB	0.39 (0.02)ˆB	0.53 (0.01)ˆA	18		^∗∗∗^
Root N_mass,1-5_ [mg g^-1^]	13 (1)	14 (1)	12 (1)	13		
Root C:N_1-5_ [g g^-1^]	30 (2)	31 (3)	37 (3)	18		
Fine root longevity^a^ [yr]	1.0 (0.3)	0.8 (0.1)	0.9 (0.1)	48		

*Absorptive roots are defined as root orders 1+2, transport roots as root orders 3–5. Values in parentheses are SE. Significant differences between the three tree groups are indicated by different upper case letters. The coefficient of variation (CV) describes trait dissimilarity. CVs larger than 50% are written in bold. Asterisks denote a significant effect of the mycorrhizal association type (AM vs. ECM) or phylogenetic group (gymnosperm vs. angiosperm) on the respective trait according to mixed effects models. Significance is indicated as (^∗^) *P* ≤ 0.1, ^∗^*P* ≤ 0.05, ^∗∗^*P* ≤ 0.01, ^∗∗∗^*P* ≤ 0.001. ^a^, literature data.*

Aboveground, gymnosperms differed by lower SLAs (83 vs. 119–148 cm^2^ g^-1^) and wood densities (470 vs. 598–653 kg m^-3^) from the hardwood species (**Table 1**). As a consequence of the difference in their leaf xeromorphic structure and ecological function, phylogenetic group affiliation had a significant effect on leaf longevity, which distinguished from the other traits by the distinctly highest coefficient of variation (149%). By contrast, despite its moderately high coefficient of variation (48%), mean fine root longevity did not significantly differ between phylogenetic groups. Further, root N_1-5_ and root C:N_1-5_ of the first to fifth root order varied only little between the investigated tree species (13–18%) and did not significantly differ between phylogenetic groups.

The phylogenetic signal estimated by the correlation between the phylogenetic distance and the trait distance matrices was highly significant for the first PCA axis calculated for root traits (PCA Root 1), which was mainly related to tissue density_1+2_, SRL_1+2_, and root diameter_1-5_ (**Table 2**). About 56% of the variation of the trait distance matrix for PCA Root 1 was explained by the relatedness of tree species (*R* = 0.75), with 6% of the nodes of the phylogenetic tree exhibiting significant trait conservatism toward PCA Root 1 (divergence SD 0.35, mean age 29% branch length of the total phylogenetic distance) and no significant divergence. Another strong phylogenetic signal was detected for the first PCA axis calculated for leaf traits (PCA Leaf 1), which was mainly related to SLA and leaf longevity (explained variation: 37%, *R* = 0.61), and a slightly weaker signal in the second axis for leaf traits (PCA Leaf 2), which was mainly related to leaf N_mass_ (explained variation: 25%, *R* = 0.50). Both, the second PCA axis for root traits (PCA Root 2; related to the root branching ratio, root C:N_1-5_, and root N_1-5_) and the two PCA axes for stature traits were not significantly influenced by a phylogenetic signal.

**Table 2:** Phylogenetic signal estimated by the correlation between the phylogenetic distance and the trait distance matrices (Mantel permutation test).

Trait complex	*R*	*P*
Leaves, PCA axis 1 (SLA and leaf longevity)	**0.61**	**0.003**
Leaves, PCA axis 2 (Leaf N_mass_)	**0.50**	**0.01**
Stature, PCA axis 1 (Max. tree height and age)	-0.07	0.53
Stature, PCA axis 2 (Wood density)	0.27	0.08
Roots, PCA axis 1 (Tissue density_1+2_,	**0.75**	**<0.001**
SRL_1+2_, and root diameter_1-5_)
Roots, PCA axis 2 (Branching ratio, root	-0.03	0.49
C:N_1-5_, and root N_mass,1-5_)

*The trait distance matrices were based on principal components derived for leaf, stature, and fine root traits of 13 Central European tree species (*cf.* Supplementary Table 3). Significant correlations (*P* ≤ 0.05) are in bold type.*

### Influence of the Mycorrhizal Association Type on Root and Leaf Traits

The mycorrhizal association type (AM and ECM) had a significant effect on the branching intensity of root systems: AM angiosperm root systems had a lower branching intensity than root systems of ECM angiosperms (5 vs. 10 tips cm^-1^; **Table 1**). The coefficient of variation between tree species for root branching intensity was moderately high, since it was not only influenced by the mycorrhizal association type but also by the phylogenetic group (the lowest branching intensity was found in ECM gymnosperms: 3 tips cm^-1^). Trees of differential mycorrhizal association type also differed in their maximum tree age, where AM angiosperms had a significantly lower life expectancy than ECM angiosperm and ECM gymnosperm tree species (230 vs. 390–415 years). The CCA discriminated between AM and ECM tree species mainly by the root traits branching ratio and branching intensity, which explained together 23% of the total variation (**Figure 2**). The Mantel permutation test highlighted that there was no phylogenetic signal in the root branching ratio (**Table 2**).

**FIGURE 3:**
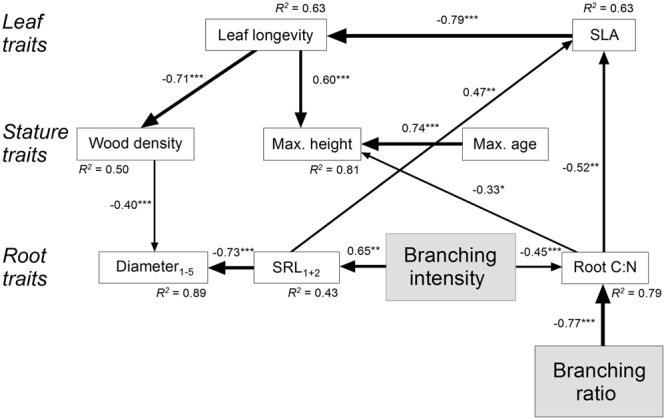
**Structural equation model (χ^2^ = 44.4, *df* = 33, *P* = 0.09) on the effect of fine root branching ratio and branching intensity for leaf, stature, and root traits of major Central European tree species.** The direction of the arrows indicates the direction of the influence; the line width illustrates the strength of the path. Path coefficients are standardized regression weights. The square of the coefficient of multiple correlations *R*^2^ is indicated at each variable. Regression weights of latent variables are fixed at unity. Significance is indicated as ^∗^*P* ≤ 0.05, ^∗∗^*P* ≤ 0.01, and ^∗∗∗^*P* ≤ 0.001. Insignificant paths and variables (leaf N_mass_, root N_mass,1-5_, fine root longevity, and tissue density_1+2_) were eliminated from the final SEM. Data for leaf longevity are log-transformed.

We chose an SEM approach to calculate complex path models of all hypothesized direct as well as indirect effects of root branching on leaf, stature, and root traits (Supplementary Figure 2). From our previous analyses (see above) we assume that branching ratio and branching intensity can be considered as indication of the mycorrhizal colonization type (*cf.* Brundrett, 2002; Smith and Read, 2008; Comas and Eissenstat, 2009; Comas et al., 2014; Eissenstat et al., 2015). Since leaf C:N and leaf longevity were closely related to each other (*R* = 0.92, *P* < 0.001) and leaf C:N was only little variable, only leaf longevity entered the model. Subsequently, all insignificant paths and variables were eliminated from the primary SEM. The final SEM (χ^2^ = 44.4, *df* = 33, *P* = 0.09) explained approximately 90% of the variation in root diameter_1-5_, 80% of the variation in root C:N, and 45% of the variation in SRL_1+2_ (**Figure 3**). The root branching ratio and intensity directly influenced root C:N (standardized direct negative effects SDE: -0.77 and -0.45). Among the strongest indirect effects of the branching ratio were its effects on SLA (standardized indirect positive effect SIE: 0.40) and leaf longevity (negative SIE: -0.32), i.e., on two aboveground leaf traits. The branching intensity had also a direct influence on SRL_1+2_ (positive SDE: 0.65). Consequently, the strongest indirect effects of the branching intensity were two-directional, on belowground root diameter_1-5_ (negative SIE: -0.59) and on aboveground SLA (positive SIE: 0.54).

## Discussion

The LES describes the return on investment in leaves and is thought to better describe the leaf economic variation at the global scale than groupings of plant species into plant functional types (Wright et al., 2004). While the LES has been successfully translated into a WES (Chave et al., 2009), the transfer into a globally consistent RES is still inconsistent. In the current study we could not identify an RES in analogy to the LES. We found two main root trait dimensions that were either influenced by phylogeny (root morphology of lower order root traits) or by root branching. Root branching was also the leading belowground trait that indirectly influenced (via root C:N) several aboveground traits.

### The LES is Not Mirrored Belowground

The physical, chemical, and biological selection pressures for leaves and roots are vastly different. Soil resource uptake, i.e., water and nutrient uptake, is constrained among others by (soil) climatic conditions, diffusion barriers, the soil matrix, bedrock chemistry, pore size, and soil compaction. Yet in a simplification of environmental conditions and constraints acting on leaves and roots, the RES is explored as analog of the aboveground trait axis between SLA, leaf N content per unit mass, rates of respiration and photosynthesis, growth rate, and longevity (Reich et al., 1997). According to this chain of thought, SRL should have a key position in the RES similar to SLA in the LES, and correlate positively with root N and respiration and negatively with root longevity. Empirical evidence for such RES is contradictory, though. In our study some leaf traits of the investigated tree species were reflected by their root counterparts (surface areas and C:N) while others central to the RES were not (N content per unit mass and longevity; Supplementary Table 4). Previously, SLA and SRL as well as leaf and root N and P contents were found positively related across tree species (Reich et al., 1998; Withington et al., 2006; Holdaway et al., 2011), but the generality of the coordinated variation of above- and belowground morphological and chemical traits has been challenged (Valverde-Barrantes et al., 2015; Weemstra et al., 2016). Further, the limited number of studies which have compared the root longevities of different tree species seem to indicate that leaf and root lifespans are generally uncorrelated (Withington et al., 2006; Espeleta et al., 2009; McCormack et al., 2012).

In addition to the missing coordinated variation of above- and belowground traits, we could not identify an RES with respect to relations between SRL or root diameter with root chemistry (root N_mass_) or function (root longevity). Root N concentration was also not correlated with morphological traits in other studies comparing different temperate tree species (Withington et al., 2006; Comas and Eissenstat, 2009; Chen et al., 2013; Valverde-Barrantes et al., 2015), which was explained by the greater cross-species variation in root morphology than in root N (Comas and Eissenstat, 2009; Chen et al., 2013; this study). Root N content of lower-order roots of temperate trees is generally less variable than its morphology since it is mainly located in cortical tissues which have a relatively constant proportion across roots of different diameter (Guo et al., 2008). Studies in different biomes have found that root N and SRL of trees correlated with root respiration (Reich et al., 1998, 2003; Chen et al., 2010), and all correlated with root lifespan (Withington et al., 2006; McCormack et al., 2012; Reich, 2014). However, a literature review and meta-analysis found little evidence for a relationship between root N and N uptake rates, which was explained by the fact that N uptake rates are less limited by the number of nutrient uptake transporters (which contain only a small fraction of N) than by the availability of N in the soil matrix and the extension of the mycorrhizal hyphae (Weemstra et al., 2016). These authors could also not reveal a consistent evidence for an RES mirroring an LES and argued that the reason is that root traits are under multidimensional restrictions: root traits are simultaneously constrained by various environmental drivers not necessarily related to resource uptake, function differently than aboveground traits, and are offset by mycorrhizal interactions (Weemstra et al., 2016). In conclusion, the key functional traits determining uptake acquisition of belowground resources may not be included in the current RES analogy of LES. Conceivable root traits for soil resource acquisition are the number of superficial adventitious roots, length and density of root hairs or hyphae, cluster root formation, and rooting depth, which relate to the branching of the root system and its rooting density in the soil.

### Root Morphology is Phylogenetically Selected

Both, the mixed variance-covariance model and the Mantel permutation test revealed a significant phylogenetic signal (as an indication of selective pressure) for morphological root traits, i.e., for root tissue density_1+2_, root diameter_1-5_, and SRL_1+2_. Root diameter was also the most important root trait discriminating between gymnosperm and angiosperm tree species in the CCA (higher mean root diameter in gymnosperms: 0.53 mm; angiosperms: 0.39 mm). The higher root diameter in gymnosperms than in angiosperms can be explained by anatomical differences in their xylem where more tracheids in gymnosperm roots are needed to achieve a similar transport capacity as in angiosperm vessels (Sperry et al., 2006). But the systematic difference in root diameter between gymnosperms and angiosperms may also give an indication of the divergence time for these morphological root traits and be explanation for the significant conservatism in these traits: the emergence of colder and drier climate during the mid to late Cretaceous has been hypothesized as a cause of adaptation and root trait diversity in angiosperms (Comas et al., 2012; Chen et al., 2013; Zanne et al., 2014); increases in SRL and tissue lignification and decreases in diameter probably increased the efficiency of root systems in an environment with lower N availability, slower decomposition rates, and adverse climatic conditions (Pittermann et al., 2012; Chen et al., 2013). Our study has shown that SRL_1+2_ had comparably high cross-species variability despite the conservatism of the root morphology trait complex (38%; **Table 1**), which may be indication of its plasticity toward different environmental conditions.

A high root diameter and a long root lifespan are considered as conservative root traits which are often assigned to conifer trees independent from their leaf habit: in a common garden experiment with different tree species, the deciduous conifer *Larix decidua* had acquisitive leaf traits, i.e., high SLA, high leaf N content, and short leaf lifespan, similar to the deciduous broadleaf trees, but conservative root traits similar to the other evergreen conifers (Withington et al., 2006). Our study only partly confirmed the classification of root traits of *L. decidua* to the conservative trait spectrum, as it resembled the conservative root characteristics of the other conifer species with respect to its root diameter_1-5_, SRL_1+2_, and tissue density_1+2_, but not with respect to root N content and root lifespan. Root N content and lifespan were generally root traits not discriminating between broadleaf trees and conifers.

Earlier studies have also found that common ancestry has strong impact on root traits such as diameter and tissue density (Comas and Eissenstat, 2009; Chen et al., 2013; Kong et al., 2014): it was concluded that ecological filtering acts stronger on leaf than on root traits (Ackerly and Reich, 1999; Reich et al., 2003; Whitman and Aarssen, 2010; Valverde-Barrantes et al., 2015) and that this is the reason why the RES (with SRL and root diameter as the key traits) is stronger supported by data collected from more closely related than from more distant tree species (e.g., Comas and Eissenstat, 2009; McCormack et al., 2012; Weemstra et al., 2016). Our study does not fully support this conclusion since we found (i) no impact of common ancestry on root architecture, but (ii) significant phylogenetic signal in leaf morphology (SLA), longevity, and chemistry (leaf N_mass_) - yet even though with lower correlation coefficients than for root morphology (*R* = 0.50–0.61 vs. 0.79). The missing phylogenetic signal in the branching ratio of the root system hints to a stronger impact by the environment and ecological filtering on root branching than by common ancestry.

### Increased Root Branching is a Response to the Environment

The root branching ratio was not influenced by phylogeny in our study. Root branching patterns are thought to largely affect root functioning (Pregitzer, 2002; Guo et al., 2008): the branching ratio of first to second order roots gives an indication of the plasticity of the absorptive root system to proliferate into locally or temporarily resource-rich patches (Hodge, 2004; Kong et al., 2014). In a study with subtropical forest species, the branching intensity and ratio showed weak phylogenetic conservatism, and were negatively correlated with soil P and N contents, suggesting that higher branching intensity may be required at low-fertility sites (Kong et al., 2014). Increased root branching is typical for ECM fungal associations (Brundrett, 2002; Smith and Read, 2008; Comas and Eissenstat, 2009; Comas et al., 2014; Eissenstat et al., 2015), which are occurring in ecosystems dominated by organic nutrients and comparably low fertility (Phillips et al., 2013). By contrast, the colonization with AM fungi has only subtle effects on root architecture (Maherali, 2014), even though it can significantly change root diameter (Comas et al., 2012; Kong et al., 2014). This difference in root architecture between ECM and AM roots was confirmed by our study when comparing only angiosperm tree species: ECM trees had a slightly lower branching ratio of first to second order roots than AM trees, but much higher branching intensity (root tips per lower order root length), i.e., ECM root tips were more clustered. However, while the branching intensity of the investigated angiosperm tree species was significantly influenced by the mycorrhizal association type and was next to the branching ratio the key trait discriminating AM from ECM angiosperm tree species, it was also a secondary factor for the discrimination between angiosperm and gymnosperm root traits. Soil nutrients in gymnosperm forests are nearly homogenously distributed due to the accumulation of their recalcitrant leaf litter over many years (Chen et al., 2016), which decreases the importance of root proliferation and leads to the lower branching intensity in gymnosperms as observed in our study.

The influence of the mycorrhizal association type on root branching is also the reason for the missing phylogenetic signal in this root trait: the mycorrhizal association type is not related to the phylogenetic relatedness of the tree host, but in contrast is phylogenetically highly diverse, both with respect to the plant host (particularly AM) and the fungal symbiont (particularly ECM). The ancestral AM symbiosis has been stably inherited since its establishment, but there have been many independent conversions of AM to ECM symbioses (>>12 independent origins) in derived lineages of some major plant clades (Brundrett, 2002; Wang and Qiu, 2006). These independent conversions were probably a consequence of the emergence of new lineages in fungi and plants as an adaptation to a change in the environment to more seasonal and arid climate approximately 135 MYA (Malloch et al., 1980; Moyersoen, 2006) and to nutrient-poorer environments. Due to their saprotrophic capabilities, ECM fungi can access recalcitrant nutrient pools that are inaccessible to AM fungi (Chalot and Brun, 1998; Blum et al., 2002; Courty et al., 2010) and, thus, are better adapted to nutrient deficiency. Increased root branching of ECM trees adds to this by supporting the proliferation and nutrient uptake from locally or temporarily resource-rich patches in the nutrient-poor ECM ecosystems.

Increased branching is a measure for a higher proportion of lower order roots with presumed fast respiration rates (Rewald et al., 2014) and high resource uptake activity (Guo et al., 2008; Rewald et al., 2011; McCormack et al., 2015). Our SEM revealed significant negative direct effects of both root branching ratio and intensity on root C:N, and negative indirect effects on SLA and leaf longevity, which may give hint on a whole plant economics spectrum with root branching as the key trait: the fast, acquisitive strategy of nutrient uptake is characterized by intensive root branching in resource-rich patches and corresponds with a tight root C:N (*viz.* relatively low C and high N content, which may be explained by lower suberin content of first order roots and faster N uptake rates), high SLA which is favorable for fast C uptake, and short leaf longevity, while the slow spectrum is characterized by the opposite set of traits. Additionally, intensive root branching also increases SRL of the pool of first and second order roots and decreases the average and lower order root diameter, which are both thought to be essential traits for fast resource acquisition.

In a comparison of the two major mycorrhizal association types in temperate forests, AM tree species have been proposed as fast in comparison to ECM species, due to the more rapid colonization of AM hyphae into N-rich patches (Hodge and Fitter, 2010), the faster turnover and decomposition of AM hyphal, root, and leaf litter (Read and Perez-Moreno, 2003; Hobbie et al., 2006; Anderson and Cairney, 2007), and the quicker soil nutrient cycling rates (Vesterdal et al., 2012; Phillips et al., 2013). The current study makes clear that part of the fast/slow trait difference between AM and ECM tree species is also due to the occurrence of gymnosperms in the ECM association type in temperate regions, which can be assigned to the conservative (slow) trait family. When considering only angiosperms, deciduous ECM trees have to be rather assigned to the acquisitive trait family, since they have significantly higher root branching intensity and higher SLA, but do not differ significantly from AM trees with respect to their root C:N or leaf longevities. This classification to the fast/slow trait spectrum does not relate to the absolute growth rates of trees though, as the majority of the investigated AM species were early successional, fast-growing species, while the majority of the ECM angiosperms were late-successional, slow-growing species, which become dominant at later stages of ecosystem succession. But the dominance of these latter species, i.e., of European beech, is probably due to better resource exploitation both aboveground (highest SLA) and belowground (comparably high root branching ratio and intensity).

## Conclusion

We conclude that root branching relates to the mycorrhizal association type and to precision foraging into resource-rich patches and, thus, is a key belowground trait that influences resource uptake rates and function, which should be central to a revised root or whole plant economics spectrum. The dominating phylogenetic signal in root morphology, i.e., on SRL and root diameter, makes morphological traits less plastic and therefore less central for the description of economics spectra of temperate tree species across different environments - even though they may be useful for the separation of functional groups. Current investigations of the RES may have been inconclusive so far since they focused on those root traits which were in analogy to the LES, but may have disregarded the key functional trait for belowground resource acquisition. Inclusion of root branching as leading root trait of a whole plant economic spectrum may greatly improve modeled growth response of forest communities to environmental change.

## Author Contributions

IM and RL conceived and designed the research project. KA and RL performed research. IM and KA analyzed the data. IM and RL wrote the manuscript. All authors approved the final version of the manuscript.

## Conflict of Interest Statement

The authors declare that the research was conducted in the absence of any commercial or financial relationships that could be construed as a potential conflict of interest.
