# Supplementary material for: Root Branching Is a Leading Root Trait of the Plant Economics Spectrum in Temperate Trees
Source: Front Plant Sci. 2017 Mar 8;8:315. doi: 10.3389/fpls.2017.00315 (PMC5340746; doi:10.3389/fpls.2017.00315)
Supplement: Supplementary file 1 [file Data_Sheet_1.PDF]

Supplementary Material

Root branching is a leading root trait of the plant economics spectrum in temperate trees

Rebecca Liese [1], Katrin Alings [1], Ina C. Meier [1*]

[1]Plant Ecology, Albrecht-von-Haller Institute for Plant Sciences, University of Göttingen, Göttingen, Germany

*** Correspondence:** Ina C. Meier; imeier1@uni-goettingen.de

1 Supplementary Figures and Tables

1.1 Supplementary Figures

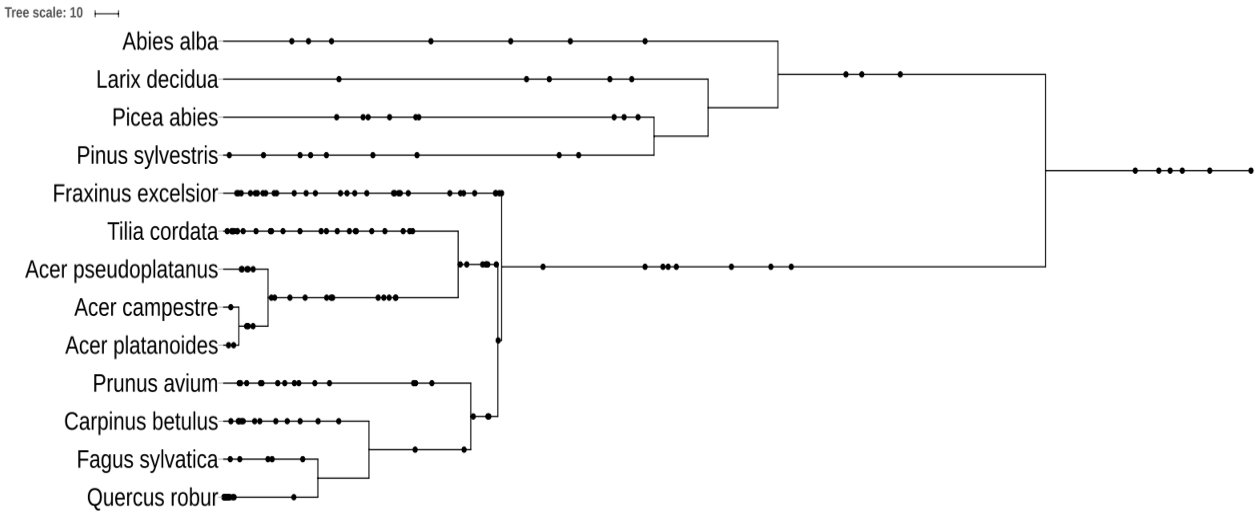

Supplementary Figure 1. Phylogenetic tree of 13 Central European tree species based on Zanne *et al.* (2014).

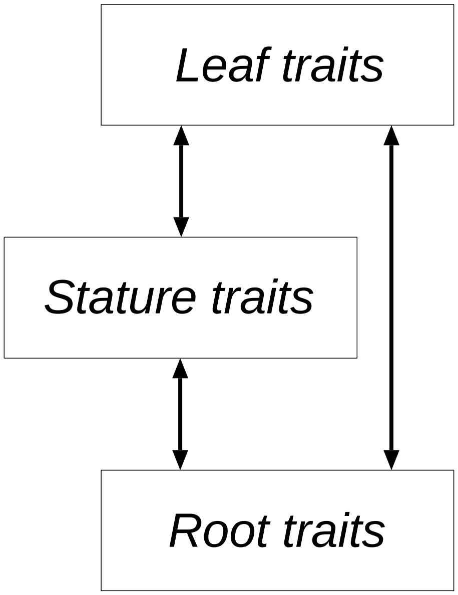

Supplementary Figure 2. Conceptual model on plausible interactions between root, stature, and leaf traits of temperate tree species.

Supplementary Table 1. Taxonomy, phylogenetic group, leaf habit, and mycorrhizal association type of the 13 major Central European tree species of this study.

Species	Code	Common name	Family	Phylogenetic group	Leaf habit	Mycorrhizal association
Pinus sylvestris	*Pisy*	Scots pine	Pinaceae	Gymnosperm	evergreen	ECM
Larix decidua	*Lade*	European larch	Pinaceae	Gymnosperm	summer-green	ECM
Acer campestre	*Acca*	Field maple	Sapindaceae	Angiosperm	summer-green	AM
Prunus avium	*Prav*	Wild cherry	Rosaceae	Angiosperm	summer-green	AM
Fraxinus excelsior	*Frex*	European ash	Oleaceae	Angiosperm	summer-green	AM
Quercus robur	*Quro*	Pedunculate oak	Fagaceae	Angiosperm	summer-green	ECM
Acer platanoides	*Acpl*	Norway maple	Sapindaceae	Angiosperm	summer-green	AM
Acer pseudoplatanus	*Acps*	Sycamore maple	Sapindaceae	Angiosperm	summer-green	AM
Carpinus betulus	*Cabe*	European hornbeam	Betulaceae	Angiosperm	summer-green	ECM
Picea abies	*Piab*	Norway spruce	Pinaceae	Gymnosperm	evergreen	ECM
Abies alba	*Abal*	Silver fir	Pinaceae	Gymnosperm	evergreen	ECM
Tilia cordata	*Tico*	Little-leaved lime	Malvaceae	Angiosperm	summer-green	ECM
Fagus sylvatica	*Fasy*	European beech	Fagaceae	Angiosperm	summer-green	ECM

Supplementary Table 2. Trait values of the 13 major Central European tree species of this study. Succession status: e = early, m = mid, l = late. Mycorrhizal association type: AM, arbuscular mycorrhiza; ECM, ectomycorrhiza. Phylogenetic group: A, angiosperm; G, gymnosperm. [a], literature data. n/d, no data.

	Pisy	*Lade*	*Acca*	*Prav*	*Frex*	*Quro*	*Acpl*	*Acps*	*Cabe*	*Piab*	*Abal*	*Tico*	*Fasy*
**Succession status**	**e**	**e**	**e**	**e**	**e-m**	**m**	**m-l**	**m-l**	**m-l**	**l**	**l**	**l**	**l**
**Mycorrhizal association**	**ECM**	**ECM**	**AM**	**AM**	**AM**	**ECM**	**AM**	**AM**	**ECM**	**ECM**	**ECM**	**ECM**	**ECM**
**Phylogenetic group**	**G**	**G**	**A**	**A**	**A**	**A**	**A**	**A**	**A**	**G**	**G**	**A**	**A**
Leaves													
SLA [cm^2 g^{-1}]	57	126	127	146	103	121	113	105	134	88	62	167	169
Leaf N_{mass} [mg g^{-1}]	14	31	17	16	20	19	22	22	20	27	18	25	23
Leaf C:N [g g^{-1}]	45	26	27	28	23	24	22	21	24	n/d	n/d	19	21
Leaf longevity[a] [yr]	3.7	0.5	0.5	0.5	0.4	0.5	0.5	0.5	0.6	7.1	8.2	0.4	0.5
Stature													
Max. tree height[a] [m]	50	50	15	25	49	60	40	40	30	70	68	45	56
Wood density[a] [kg m^{-3}]	490	550	610	550	650	650	590	590	790	430	410	490	680
Max. tree age[a] [yr]	450	450	100	150	300	500	300	300	250	300	450	400	450
Roots													
SRL_{1+2} [m g^{-1}]	26	28	44	60	29	55	37	76	54	25	22	52	53
Tissue $density_{1+2}$ [g cm^{-3}]	0.23	0.22	0.17	0.19	0.21	0.16	0.21	0.12	0.17	0.26	0.24	0.15	0.13
Branching ratio [n n^{-1}]	1.6	2.9	2.4	2.7	3.5	1.9	3.0	2.7	2.3	2.6	2.8	2.2	2.8
Branching intensity [tips cm^{-1}]	4.3	1.8	8.5	4.5	1.8	12.9	4.9	7.3	8.4	5.5	1.8	8.7	8.6
Absorptive : transport roots [g g^{-1}]	0.3	1.0	0.7	0.5	2.1	0.4	1.2	0.7	0.6	0.7	0.8	0.4	0.7
Root $diameter_{1+2}$ [mm]	0.46	0.46	0.41	0.36	0.46	0.42	0.42	0.39	0.38	0.44	0.50	0.43	0.44
Root $diameter_{1-5}$ [mm]	0.53	0.52	0.43	0.34	0.46	0.36	0.40	0.34	0.34	0.53	0.54	0.45	0.40
Root $N_{mass, 1-5}$ [mg g^{-1}]	10	13	13	12	15	12	13	14	12	12	13	16	16
Root $C:N_{1-5}$ [g g^{-1}]	46	33	27	36	25	38	31	32	33	35	32	27	26
Fine root longevity[a] [yr]	0.7	1.1	1.7	0.3	0.6	1.0	1.6	1.7	0.8	0.7	1.1	0.6	0.6

Supplementary Table 3. Three individual principal components analyses (PCA) for the identification of major leaf, stature, and root trait gradients, respectively, among the 13 tree species. The most characteristic variables (according to their loading) of each PCA axis are in bold type.

	**PCA axis 1**	**PCA axis 2**
**Leaf PCA**	**Leaf 1**	**Leaf 2**
Eigenvalue	0.590	0.325
Explained variation (cumulative)	59.0	91.5
SLA	**-0.933**	-0.132
Leaf N_{mass}	-0.486	**0.868**
Leaf longevity	**0.823**	0.427
**Stature PCA**	**Crown 1**	**Crown 2**
Eigenvalue	0.880	0.09
Explained variation (cumulative)	88.0	97.0
Max. tree height	**-0.969**	0.170
Wood density	0.331	**-0.880**
Max. tree age	**-0.976**	-0.178
**Root PCA**	**Root 1**	**Root 2**
Eigenvalue	0.491	0.350
Explained variation (cumulative)	49.1	84.1
SRL_{1+2}	**-0.927**	0.187
Tissue $density_{1+2}$	**0.962**	-0.006
Branching ratio	0.063	**-0.912**
Branching intensity	-0.748	0.459
Root $diameter_{1-5}$	**0.859**	-0.156
Root $N_{mass, 1-5}$	-0.454	**-0.812**
Root $C:N_{1-5}$	0.410	**0.841**
Fine root longevity	-0.042	-0.022

Supplementary Table 4. Pearson's correlation matrix between leaf and fine root traits. Only significant correlations are shown. Significance is indicated as (*) $P \leq 0.1$, * $P \leq 0.05$, and ** $P \leq 0.01$.

Root trait	Root order	SLA	Leaf N_{mass}	Leaf C:N	Log (leaf longevity)
SRL	1+2	0.59*			-0.62*
	1-5			-0.58(*)	-0.66*
SRA	1+2	0.61*		-0.54(*)	-0.63*
	1-5			-0.59*	-0.61*
Diameter	1+2	-0.51(*)			0.55*
	1-5	-0.56*			0.71**
Tissue density	1+2	-0.68*		0.61*	0.70**
	1-5				
Branching ratio	1+2			-0.56(*)	
Branching intensity	1+2	0.49(*)			
Absorptive : transport roots	1-5				
Root N_{mass}	1-5	0.56*		-0.78**	
Root C:N	1-5	-0.53(*)		0.80**	
Log (fine root longevity)					
